# The cyclin dependent kinase inhibitor Roscovitine prevents diet-induced metabolic disruption in obese mice

**DOI:** 10.1038/s41598-021-99871-z

**Published:** 2021-10-13

**Authors:** Nabil Rabhi, Kathleen Desevin, Briana Noel Cortez, Ryan Hekman, Jean Z. Lin, Andrew Emili, Stephen R. Farmer

**Affiliations:** 1 Department of Biochemistry, Boston University School of Medicine, 72 East Concord Street, Boston, MA 02118 USA; 2 Center for Network Systems Biology, Boston University School of Medicine, 72 East Concord Street, Boston, MA 02118 USA

**Keywords:** Endocrinology, Endocrine system and metabolic diseases, Obesity

## Abstract

Most strategies to treat obesity-related disorders have involved prevention of diet-induced weight gain in lean mice. Treatment of obese individuals will require therapies that reverse the detrimental effects of excess body weight. Cyclin-dependent kinases have been shown to contribute to obesity and its adverse complications. Here, we show that roscovitine; a an orally available cyclin-dependent kinase inhibitor; given to male mice during the last six weeks of a 19-week high fat diet, reduced weight gain and prevented accompanying insulin resistance, hepatic steatosis, visceral adipose tissue (eWAT) inflammation/fibrosis as well as restored insulin secretion and enhanced whole body energy expenditure. Proteomics and phosphoproteomics analysis of eWAT demonstrated that roscovitine suppressed expression of peptides and phosphopeptides linked to inflammation and extracellular matrix proteins. It also identified 17 putative protein kinases perturbed by roscovitine, including CMGC kinases, AGC kinases and CAMK kinases. Pathway enrichment analysis showed that lipid metabolism, TCA cycle, fatty acid beta oxidation and creatine biosynthesis are enriched following roscovitine treatment. For brown adipose tissue (BAT), analysis of upstream kinases controlling the phosphoproteome revealed two major kinase groups, AGC and CMGC kinases. Among the top enriched pathways were insulin signaling, regulation of lipolysis in adipocytes, thyroid hormone signaling, thermogenesis and cAMP-PKG signaling. We conclude that roscovitine is effective at preventing prolonged diet-induced metabolic disruption and restoring mitochondrial activity in BAT and eWAT.

## Introduction

Obesity has reached pandemic proportions contributing to the development of type 2 diabetes mellitus, and other metabolic disorders, such as dyslipidemia, liver dysfunction and cardiovascular disease^1, 2^. The incidence of these disorders is expected to double by 2030 and expenditure of associated healthcare will exceed 100 billion in the United States alone^3, 4^. In obese individuals, white adipose tissue (WAT) responds to caloric excess through the enlargement of existing white adipocytes (hypertrophy) and the recruitment of new fat cells (hyperplasia). Whereas hyperplasia is often associated with metabolically healthy obesity, hypertrophy is a hallmark of unhealthy obesity and correlates with greater inflammation and insulin resistance^5^. Obese WAT undergoes substantial remodeling, which involves expansion of a host of stromal cells as well as deposition of extracellular matrix (ECM)^5, 6^. The adipocytes become hypoxic due to their large size and a reduction in angiogenesis resulting in production of profibrogenic factors such as collagen V1a3 and endotrophin^7–10^. In mice and humans, fibrosis is a hallmark of obesity that is tightly associated with inflammation and characterized by immune cell infiltration^11, 12^. Morbidly obese individuals have a high degree of fibrosis in both subcutaneous and visceral AT, with visceral fibrosis being associated with elevated serum triglycerides and limited adipocyte size, while subcutaneous fibrosis is negatively associated with weight loss after bariatric surgery^13, 14^. This obesity-associated remodeling of adipose tissue accompanies an extensive decline in adipocyte mitochondrial activity that impacts major adipocyte functions such as adiponectin production^10, 15–17^. Consequently, therapeutic approaches to combat obesity-associated disorders should include strategies to restore mitochondrial function^18^. Although some weight-loss drugs have been developed, they often do not work well or have serious side effects^2, 19–22^. Therefore, there is an urgent need to develop new drugs for the treatment of obesity and its complications.

Cyclin-dependent kinases (CDKs) are the central regulators of cell growth, development, and proliferation^23^. In recent years, CDKs has emerged as key regulators of adipose tissue homeostasis and have been showed to be hyperactivated in obesity^24–28^. Roscovitine (Seliciclib) is an orally available cyclin-dependent kinase inhibitor^29^ and currently evaluated as a potential drug to treat cancers, neurodegenerative diseases, inflammation, viral infections, polycystic kidney disease, Cushing's disease and glomerulonephritis^30^. It also has shown multiple benefits in cystic fibrosis (CF) studies and is efficacious in preclinical arthritis models and has been found to exert anti- inflammatory and anti-fibrosis effects^31–34^. We previously showed that roscovitine induces browning in inquinal WAT through mechanisms that are consistent with-it blocking phosphorylation of PPARγ at sites shown to regulate insulin sensitivity^35^. We further demonstrated that roscovitine enhanced energy expenditure in lean mice which prevented obesity following a limited diet of high fat. In the present study, we document that roscovitine reduces weight gain in obese mice and prevents accompanying inflammation, fibrosis and insulin resistance.

## Results

### Roscovitine treatment reduces metabolic disruption in obese mice

**Figure 1:**
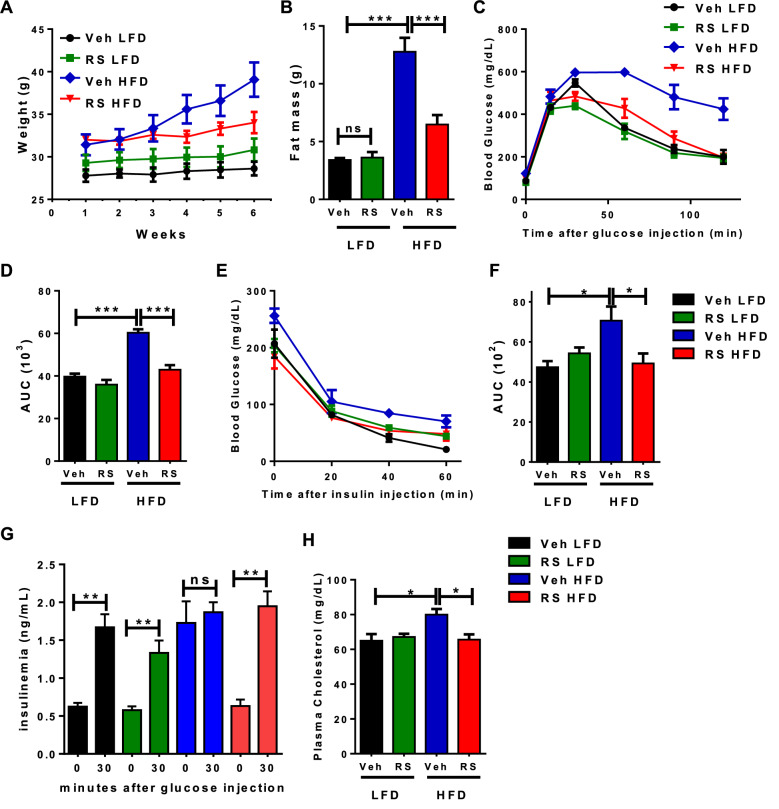
Roscovitine treatment reduces metabolic disruption in obese mice. (**A**) Body weight progression during the 6 weeks of roscovitine (50 mg/kg) or vehicle treatment starting 13 weeks of low-fat diet (LFD) or high-fat diet (HFD) feeding (n = 5 male mice/ group). (**B**) Fat mass determined by nuclear magnetic resonance (NMR). (**C**) Intraperitoneal glucose tolerance test (ipGTT) in 19–20 weeks LFD and HFD fed mice treated for 6 weeks with roscovitine or vehicle and (**D**) the corresponding area under the curve (AUC) of ipGTT (n = 5 male mice / group). (**E**) Intraperitoneal insulin tolerance test in 19–20 weeks LFD and HFD fed male mice treated for 6 weeks with roscovitine or vehicle and (**F**) the corresponding area under the curve (AUC) of ipGTT (n = 5 male mice/group). (**G**) Blood insulin levels as measured during ipGTT in LFD and HFD fed male mice treated for 6 weeks with roscovitine or vehicle (n = 5). (**H**) Fasting plasma cholesterol level in 19–20 weeks LFD and HFD fed male mice treated for 6 weeks with roscovitine or vehicle (n = 5/group). Data were analyzed and illustrated using GraphPad Prism 6.0 software (GraphPad, https://www.graphpad.com). All values are expressed as means ± SEM; *p < 0.05, **p < 0.01, and ***p < 0.001.

Previous studies demonstrated that roscovitine given to lean mice induced browning of WAT, enhanced energy expenditure and attenuated the weight gain from a 9-week high fat diet (HFD)^35^. In those studies, the mice were treated with roscovitine just 3 weeks into the HFD, at a time prior to dramatic metabolic changes. Here, we investigated whether roscovitine has the same beneficial effects when given to obese mice displaying metabolic disruption. Male mice were therefore fed either an HFD or LFD for 13 weeks and roscovitine was then administered for an additional 6 weeks. Roscovitine had no observable effect on mice fed LFD, however it prevented weight and fat mass gain in HFD fed mice without any change in lean mass (Fig. 1A,B, Supplemental Fig S1A). It also restored glucose clearance during ipGTT tests (Fig. 1C,D), insulin sensitivity (Fig. 1E,F), in vivo glucose stimulated insulin secretion (GSIS) (Fig. 1G) as well as fasting plasma circulating cholesterol (Fig. 1H). Overall, roscovitine prevented metabolic dysfunctions in obese mice without having any effect on mice under low fat diet.

### Roscovitine treatment prevents hepatic steatosis

**Figure 2:**
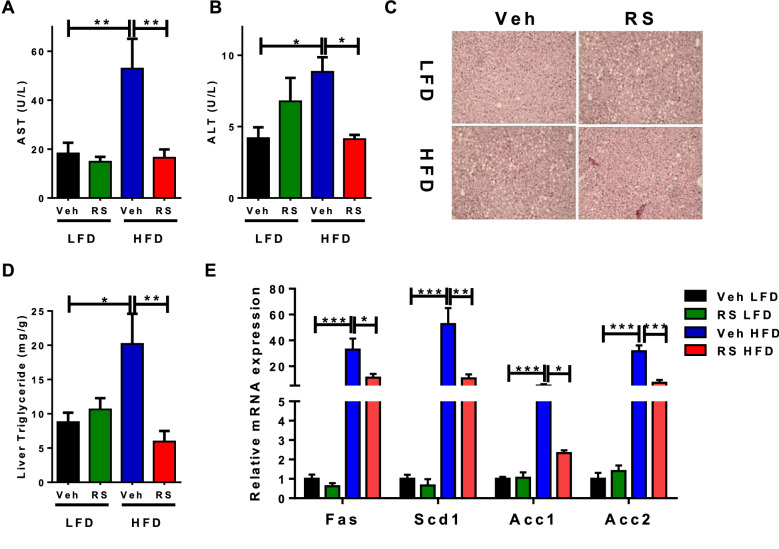
Roscovitine treatment prevents hepatic steatosis. (**A**) Serum level of aspartate aminotransferase (AST) in 19–20 weeks LFD and HFD fed male mice treated for 6 weeks with roscovitine or vehicle (n = 5). (**B**) Serum level of Alanine aminotransferase in 19–20 weeks LFD and HFD fed male mice treated for 6 weeks with roscovitine or vehicle (n = 5). (**C**) Representative pictures of H&E-stained liver cross sections in 19–20 weeks LFD and HFD fed male mice treated for 6 weeks with roscovitine or vehicle. (**D**) Fasting liver triglyceride levels in 19–20 weeks LFD and HFD fed mice treated for 6 weeks with roscovitine or vehicle (n = 5/group). (**E**) mRNA expression level of lipogenic genes in 19–20 weeks LFD and HFD fed male mice treated for 6 weeks with roscovitine or vehicle (n = 5 / group). Data were analyzed and illustrated using GraphPad Prism 6.0 software (GraphPad, https://www.graphpad.com). All values are expressed as means ± SEM; *p < 0.05, **p < 0.01, and ***p < 0.001.

The extended feeding regimen (19–20 weeks) on HFD elevated the circulating levels of liver enzymes aspartate transferase (AST) and alanine aminotransferase (ALT) reflecting damaged liver. However, both parameters were dramatically attenuated following treatment with roscovitine (Fig. 2A,B). As expected, HFD caused hepatic steatosis in vehicle treated mice as indicated by accumulation of lipid droplets, which was extensively reduced following drug treatment (Fig. 2C). Consistent with these results, the HFD-induced liver triglycerides and lipogenic (FAS, SCD1, ACC1 and ACC2) mRNA expression were reduced by roscovitine (Fig. 2D,E).

### Roscovitine prevents diet-induced hypertrophy of iWAT without activating a thermogenic program

**Figure 3:**
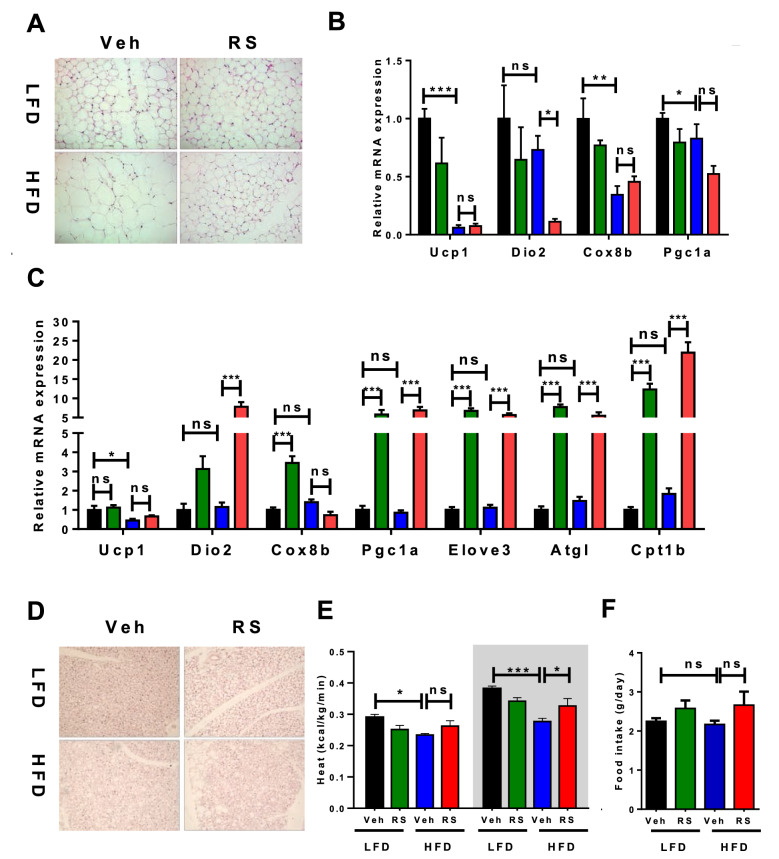
Roscovitine prevents diet-induced hypertrophy of iWAT and induces a thermogenic program in BAT without enhanced UCP1 expression. Analysis of iWAT and BAT in 19–20 weeks LFD and HFD fed male mice treated for last 6 weeks with roscovitine or vehicle. (**A**) Representative pictures of H&E-stained subcutaneous adipose tissue (iWAT) cross sections. (**B**) mRNA expression level of thermogenic genes in iWAT (n = 5 / group). (**C**) mRNA expression level of thermogenic genes in BAT (n = 5/group). (**D**) Representative pictures of H&E-stained brown adipose tissue (BAT) cross sections. (**E**) Heat production during 12 h light (left) and dark (right) cycles measured by CLAMS (n = 5 / group). (**F**) Cumulative food intake of mice in CLAMS (n = 5 / group). Data were analyzed and illustrated using GraphPad Prism 6.0 software (GraphPad, https://www.graphpad.com). All values are expressed as means ± SEM; *p < 0.05, **p < 0.01, and ***p < 0.001.

Examination of inguinal WAT cross sections stained with Hematoxylin and Eosin (H&E) revealed that roscovitine prevented the HFD increase in adipocyte size (hypertrophy), but unlike earlier studies in lean mice did not cause appearance of multilocular beige adipocytes (browning) (Fig. 3A). Furthermore, HFD suppressed expression of thermogenic genes UCP1, Cox8b and Dio2 in iWAT, which was not restored or enhanced by roscovitine (Fig. 3B). It is interesting that roscovitine did not induce browning of iWAT in mice fed LFD for 19 weeks, suggesting that obesity alone is not the suppressor of beige adipocyte formation.

### Roscovitine induces a thermogenic program in BAT without enhanced UCP1 expression

Even though roscovitine could not induce thermogenic mRNAs in iWAT of HFD mice, it did induce Elov3, Pgc1a, Cpt1b and Atgl expression in BAT in both HFD and LFD fed mice (Fig. 3C). Importantly, UCP1 mRNA was unaffected by roscovitine; in fact, HFD significantly suppressed its expression that could not be restored to LFD levels by roscovitine. This suppression of UCP1 was also accompanied by accumulation of lipids in brown adipocytes (Fig. 3D) and a decrease in whole body heat production following an HFD (Fig. 3E). Importantly, the HFD-induced drop in heat production was prevented by roscovitine without affecting food intake (Fig. 3F) or ambulatory activity (Supplemental Figs. S2A, S2B). Additionally, RER was unaffected by roscovitine under LFD and HFD conditions (Supplemental Figs. S2C, S2D). These results suggest that roscovitine restored mitochondrial activity (heat production) while redirecting lipids storage to a more active BAT. The restoration of BAT activity by roscovitine was independent of enhanced UCP1 expression, suggesting that the healthier mitochondria enabled the restoration of thermogenic activity.

### Roscovitine suppresses expression of inflammatory and fibrosis genes in eWAT of obese mice

**Figure 4:**
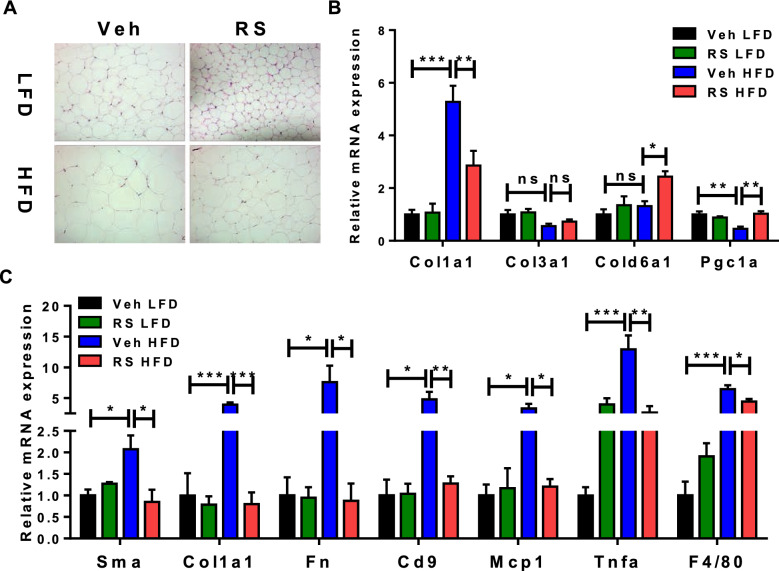
Roscovitine suppresses expression of inflammatory and fibrosis genes in eWAT. Analysis of eWAT in 19–20 weeks LFD and HFD fed male mice treated for last 6 weeks with roscovitine or vehicle. (**A**) Representative pictures of H&E-stained epididymal adipose tissue (eWAT) cross sections. (**B**) mRNA expression level of collagens subtype genes in mature adipocytes of eWAT (n = 5 / group). (**C**) mRNA expression level of fibrosis and inflamatory genes in the stromal vascular fraction of eWAT (n = 5 / group). Data were analyzed and illustrated using GraphPad Prism 6.0 software (GraphPad, https://www.graphpad.com). All values are expressed as means ± SEM; *p < 0.05, **p < 0.01, and ***p < 0.001.

Histological examination of eWAT (Fig. 4A) showed a similar HFD-induced hypertrophy of adipocytes, which was reduced by roscovitine. Interestingly, roscovitine caused a noticeable decrease of adipocyte size in mice fed LFD suggesting that it also prevents age-related obesity. As reported by us previously, the HFD induced expression of a fibroblastic-like program in the mature adipocytes, which was prevented by roscovitine (Fig. 4B). To assess other obesity-associated disorders, we measured the level of expression of inflammatory and fibrotic mRNAs in the stromal vascular fraction (SVF) of eWAT. The fibrosis (Sma, Fn, Col1a1 and CD9) and inflammatory (MCP1 and TNFa) mRNAs were increased by HFD, but remained at LFD levels following treatment with roscovitine (Fig. 4C).

### Roscovitine inhibits CDKs activity and enhances creatine synthesis in eWAT

**Figure 5:**
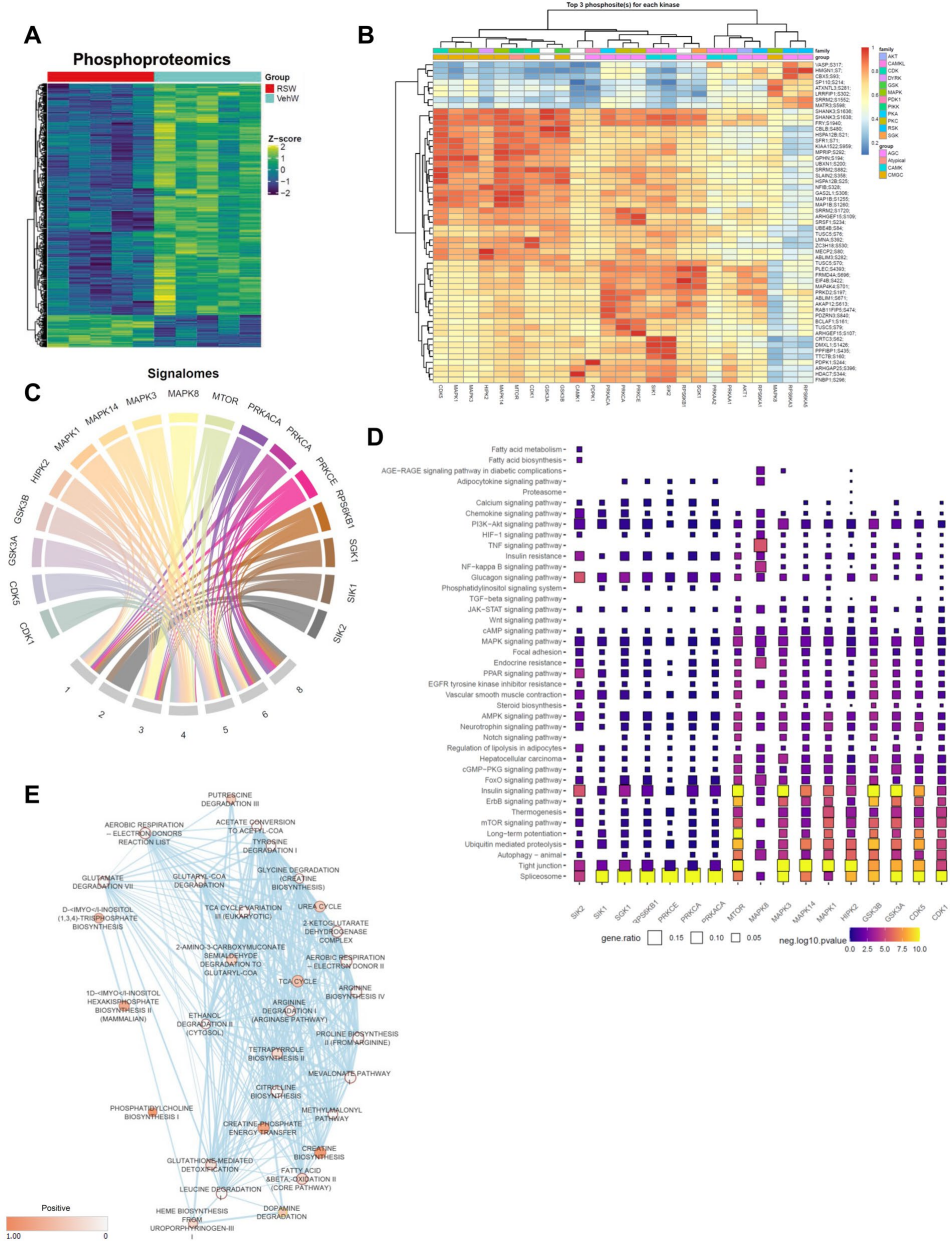
Roscovitine inhibits CDKs activity and enhances creatine synthesis in eWAT. (**A**) Heatmap of differential phosphoproteome in eWAT of 19–20 weeks LFD and HFD fed male mice treated for 6 weeks with roscovitine or vehicle (n = 5 / group). (**B**) A clustered heatmap of the combined kinase-substrate score for the top three phosphosites of all evaluated kinases. A higher combined score denotes a better fit to a kinase motif and kinase-substrate phosphorylation profile of a phosphosite. (**C**) Signalomes identified from eWAT phosphoproteome. The branching nodes consist of 17 significantly active kinases. Edges between nodes connect kinases to the protein modules they regulate. (**D**) KEGG pathway over-representation pathway analysis of kinase substrates (prediction score > 0.5). The squares are colored by negative log10 p-value (scaled across pathways) and sized by the ratio of genes present in the KEGG gene set (the larger the square, the greater the proportion of kinase substrates present in the gene set). Any pathway that is not significantly represented is depicted as a grey square. Pathways were considered to be significantly over-represented when p < 0.05. (**E**) Results of gene set enrichment analysis MOMENTA (GSEA MOMENTA) of up regulated proteins in response to roscovitine treatment in HFD mice visualized with Cytoscape Enrichment Map. Each node corresponds to a gene set either up-regulated (red) or down-regulated (blue). Edges (bleu lines) link sets with shared genes, and thickness of lines correlates with the number of genes in common between two sets. Only gene sets with FDR < 0.05 and p < 0.01 were included in visualizations. Data are illustrated using GraphPad Prism 6.0 software (GraphPad, https://www.graphpad.com).

To gain a greater understanding of processes mediating the beneficial effects of roscovitine and downstream signaling pathways affected by its treatment, we performed proteomic and phosphoproteomic profiling of eWAT from male mice fed an HFD with or without roscovitine. Proteomics analysis identified 4948 proteins and 5774 phosphosites (Fig. 5A, Supplemental Fig. S3A), of which roscovitine induced a limited set of differential proteins but suppressed expression of a larger array of polypeptides and phosphopeptides (Supplemental Figs. S3B, S3C).

Among the proteins significantly up regulated by roscovitine, 322 were annotated as proteins related to metabolism of which 111 were related to lipid metabolism. GO analysis revealed an enrichment of pathways linked to beta-oxidation and TCA cycle (Supplemental Fig. S3D).

We next computationally analyzed the identified phosphopeptides to predict the protein kinases targeted by roscovitine that are responsible for drug-induced changes in phosphorylation profiles we identified. To compare phosphorylation profiles between vehicle- and roscovitine-treated HFD mice, we applied the PhosR algorithm to differentially identified phosphosites^36^. The computational tool combines multi-step kinase-substrate scoring methods based on both annotated kinase recognition motifs and the dynamic phosphorylation profiles of specific sites. The combined scores across all kinases are then integrated and used to prioritize the kinase most likely to regulate a particular phosphosite. This phosphoproteome analysis uncovered potential kinase-substrate pairs and implicated 17 kinases perturbed by roscovitine treatment (Fig. 5B).

The kinase dendrogram shown in Fig. 5B reveals three major kinase groups governing the drug-responsive phosphoproteome (CMGC kinases [e.g., CDKs and MAPKs], AGC kinases [e.g., S6K and PKC isoforms], and CAMK kinases [e.g., SIK1 and SIK2]). In particular, CDK5 a well-established roscovitine target^29^ was among the top predicted kinases. We next generated signalome models wherein dynamic changes in phosphorylation within and across proteins were conjointly analyzed (Fig. 5C). This approach allowed for the generation of discrete protein modules with phosphosites sharing similar dynamic phosphorylation profiles and putative kinase regulation in common. This revealed seven discrete protein modules significantly changed by roscovitine, six of which were down regulated in the drug-treated mice (Fig. 5C, Supplemental Fig. S3E). As expected, modules that are predicted to be regulated by CDK5 were downregulated while the only upregulated module was deemed regulated by MAPKs. Interestingly, kinases likely contributing to the downregulated modules were previously reported to contribute to adipose tissue dysfunction during obesity such as SIK2 and PKC isoforms^37–41^. The predicted inhibition of SK6 is consistent with the overall reduction in protein level we observed by the proteomic profiling.

Pathway analysis of the kinases-substrate pairs associated with each kinase showed that the top pathways perturbed by roscovitine treatment are tight junctions, MTOR and insulin signaling (Fig. 5D). Consistent with the kinase-substrate pathways analysis, gene set enrichment analysis of the eWAT proteomics data showed that roscovitine increased proteins associated with cell–cell adhesion and tight junctions, regulation of protein secretion, and regulation of tissue remodeling (Supplemental Fig. S3F). Interestingly, pathways linked to lipid metabolism and fatty acid oxidation metabolism were also up regulated.

To further uncover the metabolic effect of roscovitine treatment, we applied the GSEA MOMENTA algorithm to both the proteome and phosphoproteome (Fig. 5E). This tool enables analysis of enriched metabolic proteins to identify likely up regulated metabolic pathways. The analysis (Supplemental Figs. S3D, 3F) revealed that terms as associated with the TCA cycle, fatty acid beta oxidation and phosphatidylcholine biosynthesis were enriched following roscovitine treatment suggesting that mitochondria become more active. Interestingly, creatine biosynthesis and creatine phosphate energy transfer were the top enriched terms; guanidinoacetate methyltransferase (GAMT), the rate limiting enzyme of creatine biosynthesis, is significantly up regulated in eWAT from roscovitine-treated mice (Supplemental Table 1) suggesting that creatine pathways combined with more active mitochondria in eWAT contribute to the roscovitine-induced weight loss.

### Roscovitine promotes the activity of brown fat

**Figure 6:**
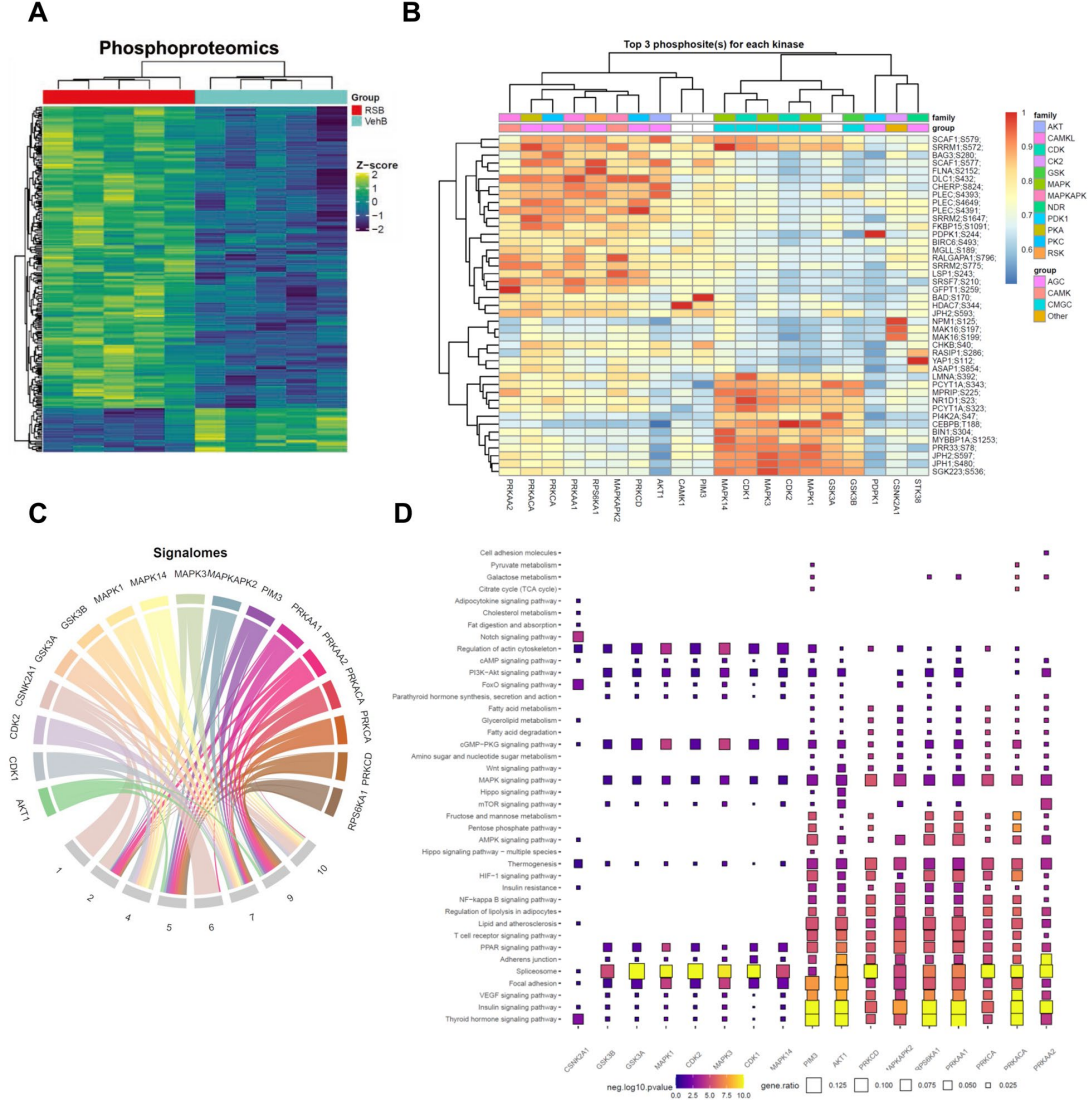
Roscovitine treatment leads to remodeling of BAT. (**A**) Heatmap of differential phosphoproteome in BAT of 19–20 weeks LFD and HFD fed male mice treated for 6 weeks with roscovitine or vehicle (n = 5 / group). (**B**) A clustered heatmap of the combined kinase-substrate score for the top three phosphosites of all evaluated kinases. A higher combined score denotes a better fit to a kinase motif and kinase-substrate phosphorylation profile of a phosphosite. (**C**) Signalomes identified from BAT phosphoproteome. The branching nodes consist of 17 significantly active kinases. Edges between nodes connect kinases to the protein modules they regulate. (**D**) KEGG pathway over-representation pathway analysis of kinase substrates (prediction score > 0.5). The squares are colored by negative log10 p-value (scaled across pathways) and sized by the ratio of genes present in the KEGG gene set (the larger the square, the greater the proportion of kinase substrates present in the gene set). Any pathway that is not significantly represented is depicted as a grey square. Pathways were considered to be significantly over-represented when p < 0.05. Data are illustrated using GraphPad Prism 6.0 software (GraphPad, https://www.graphpad.com).

We next applied the same proteomic approach to gain insight into the effect of roscovitine on BAT from HFD fed mice treated with vehicle or compound for 6 weeks. Surprisingly, we found that unlike in eWAT, roscovitine led to a significant up regulation of a large number of proteins and phosphosites in BAT (Fig. 6A, Supplemental Figs S4A, S4B, S4C). The computational analysis of upstream kinases controlling the phosphoproteome revealed two major kinase groups (AGC kinases [e.g., PKD1 and PKA] and CMGC kinases [e.g., CDKs and MAPKs] (Fig. 6B). Signalome modules analysis showed that all substrates are up regulated by roscovitine (Fig. 6C, Supplemental Fig. S4D). Among the top enriched pathways associated with the substrates associated with each kinase, we found insulin signaling, regulation of lipolysis in adipocytes, thyroid hormone signaling, thermogenesis and cAMP-PKG signaling, suggesting that roscovitine treatment led to a more active BAT (Fig. 6D). It is interesting to note that proteins associated with focal adhesion and actin cytoskeleton are also up regulated consistent with an enhanced oxidative metabolism^42^. As in eWAT, proteins associated with spliceosomes were enriched in BAT from roscovitine-treated mice. Gene set enrichment analysis of both the proteome and phosphoproteome affected by roscovitine also showed an enrichment of terms associated with mRNA processing, as well as the extracellular matrix and ATPase activity (Supplemental Fig. S4E). Overall, roscovitine treatment led to remodeling of BAT and restored its activity which was suppressed by HFD.

## Discussion

In this report, we demonstrate that roscovitine is an effective anti-obesity drug that prevents weight and fat mass gain when administered to mice during the last 6 weeks of a 19-week HFD. It additionally suppresses the diet-induced adipose inflammation, insulin resistance, and hepatic steatosis and decreases circulating levels of triglycerides and cholesterol while elevating insulin secretion. Of particular interest was its ability to counteract the fibrosis that becomes prominent in visceral AT with prolonged HFD. Proteomic analysis showed reduction in fibrotic proteins and restoration of the mitochondrial program which is extensively reduced by the prolonged diet.

We have previously shown that roscovitine induces browning of subcutaneous WAT in lean mice, but that did not occur in mice fed an HFD for several weeks. In those earlier studies, we demonstrated that roscovitine induced a beiging-associated enhancement of energy expenditure which contributed to its anti-obesity activity. Enhanced energy expenditure and/or satiety are the likely means by which roscovitine prevents the detrimental effects of the chronic diet observed in the present studies. Measurement of food intake of individual mice in metabolic cages showed no difference between vehicle or drug treated animals. The metabolic cage studies did however demonstrate an elevation in heat production (energy expenditure) in response to roscovitine treatment of HFD fed mice but not to the extent of those mice on a low-fat diet. The proteomic analysis of eWAT and BAT indicate that this is likely due to a combination of both healthier mitochondria and activation of creatine biosynthesis.

We propose that roscovitine is having a systemic effect on multiple organs most notably adipose tissue and liver that impacts metabolic homeostasis and energy expenditure. The proteomic analysis of eWAT demonstrates that roscovitine can affect several signaling pathways likely through its initial inhibition of cyclin dependent kinases particularly CDK5. Studies by Spiegelman and collaborators have previously shown that suppression of CDK5 leads to activation of ERK by preventing phosphorylation-associated inhibition of MEK1 activity^28^. These alterations in eWAT signaling however may differ in other organs giving rise to the overall impact of roscovitine on metabolic homeostasis. In BAT, for instance, roscovitine led to up regulation of large sets of proteins and phosphosites revealing involvement of two major kinase groups (AGC kinases [e.g., PKD1 and PKA] and CMGC kinases [e.g., CDKs and MAPKs] (Fig. 6B). Interestingly, PKA and p38 MAPK have been shown to play pivotal roles in activation of the thermogenic program^43, 44^.

The chronic HFD caused hepatic steatosis accompanied by an increase of liver enzymes AST and ALT, elevation of circulating levels of triglycerides and cholesterol as well as increase in expression of lipogenic genes Fas, Acc1, Scd1 and Acc2 (Fig. 2). The ability of roscovitine to suppress all of these processes could be a direct action on hepatocytes and/or indirect involving other organs/tissues. It is important to mention that roscovitine is not toxic since AST and ALT (indicators of hepatic toxicity) were suppressed by the drug. It will be interesting to identify the kinases and downstream pathways responsible for the observed beneficial effects of the CDK inhibitor. Studies have implicated CDK5 in hepatocarcinoma^45, 46^, its role in steatohepatitis is still unknown. Elevation of insulin secretion by roscovitine suggests that it has a direct action on pancreatic beta cells. In fact, several studies have identified a role for CDK5 in promoting beta cell dysfunction and inhibition of its kinase activity protects beta cells from glucotoxicity^47–51^.

Recovery from the chronic diet-induced disruption of metabolism by roscovitine likely involves alteration of several regulatory pathways in different organs with adipose tissue playing a central role. The proteomic analysis of eWAT shows that the top pathways perturbed by roscovitine involve the spliceosome, tight junctions, MTOR and insulin signaling as well as increased expression of proteins associated with cell–cell adhesion, regulation of protein secretion, tissue remodeling, lipid metabolism and fatty acid oxidation metabolism. Our MOMENTA analysis further revealed elevations in factors linked to the TCA cycle, fatty acid beta oxidation, phosphatidylcholine and creatine biosynthesis following roscovitine treatment, suggesting that the increased creatine pathways combined with more active mitochondria in eWAT may contribute to roscovitine-induced weight loss.

The proteomic data shows that roscovitine effects on metabolic pathways and actin cytoskeleton in eWAT is likely mediated by inhibition of CDK5. The importance of CDK5 in regulating adipose functions has elegantly been demonstrated by the studies of Spiegelman and coworkers^27, 28, 52^. They identified a CDK5 phosphorylation site at S273 in PPARγ, which is phosphorylated by CDK5 and other kinases including ERK in inflamed adipose tissue through the release of cytokines such as TNFα as well as fatty acids. Modification of ser273 by the kinases selectively attenuated PPARγ activity by suppressing its ability to transcribe proteins regulating insulin sensitivity including adiponectin and adipsin. It is very likely therefore that roscovitine enhances insulin sensitivity in chronically obese eWAT through similar mechanisms by inhibiting CDK5 associated S273 phosphorylation. It is important to mention that deletion of CDK5 rather than its inhibition led to insulin resistance due to the activation of ERK which could also phosphorylate S273^28^. ERK is activated because CDK5 normally phosphorylates and suppresses MEK1 activity, the upstream regulator of ERK^53^. We have previously shown that roscovitine causes MEK1-associated phosphorylation of ERK in adipose tissue^35^. We suggest that this does not lead to attenuated PPARγ activity and insulin resistance because roscovitine can also inhibit ERK activity^54^.

Ligand-activation of PPARγ has potent proinflammatory effects by inducing the differentiation of monocytes to alternative M2 macrophages through mechanisms involving direct suppression of transcription of inflammatory cytokine genes in monocytes^55–59^. In this regard, roscovitine by preventing phosphorylation of ser273 could promote these pro-inflammatory actions of PPARγ thus sharing the same beneficial properties of thiazolidinediones (TZDs).

Attenuation of fibrosis in obese mice by roscovitine could be due to its suppression of adipose inflammation since expression of fibrogenic genes is regulated in part by macrophage secreted factors such as TGFb^60^. Other studies have suggested that CDK5 can function as a mediator of mesenchymal cell fibrotic responses^61^. Specifically, Varga and associates indicate that the CDK5 activator, p35 is up-regulated in lesional skin from patients with scleroderma (SSc), and from mice with experimentally induced skin fibrosis. They showed that p35 and CDK5 mediate key effects elicited by TGF-ß, that are necessary and sufficient for fibrotic responses in mesenchymal cells. Furthermore, treatment of mice with roscovitine prevented and reversed dermal fibrosis induced by bleomycin or by TGF-ß receptor^61^. An additional study demonstrated that roscovitine by blocking the CDK5-ERK1/2-PPARγ axis^28^ attenuated tubulointerstitial fibrosis and improved renal function in diabetic rats^62^. In conclusion, the data demonstrate that roscovitine is an effective therapy for preventing metabolic disruption associated with obesity in part by activating mitochondrial functions in eWAT and BAT.

## Methods

All experiments were performed in accordance with relevant guidelines, regulations, and approval by Boston University School of Medicine’s Institutional Biosafety Committee.

### Animal experiments

C57BL6 male mice were purchased from The Jackson Laboratory at 4-week of age and acclimated for 2-week. Mice were housed in a temperature-controlled environment with a 12 h light–dark cycle and ad libitum water and diet. At 6-week of age mice were fed either low fat diet (Research diet, D12450B) or high fat diet (Research Diets, D12492) for 13 weeks. During the last 6 weeks of the diet, the mice were intraperitoneally (i.p.) injected daily with roscovitine (50 mg/kg) or vehicle. For diet studies, 6-week-old C57BL/6 N wild-type male mice were fed a diet with 60% kcal% fat (high fat diet, Research Diets, D12492) for 9 weeks. All animal studies were approved by the Boston University School of Medicine Institutional Animal Care and Use Committee.

### Metabolic phenotyping

Metabolic phenotyping experiments were performed according to the EMPRESS protocols (http://empress.har.mrc.ac.uk) as previously outlined^10, 63^. Briefly, intraperitoneal glucose (2 g of glucose per kg of body weight) and insulin (0.75 U of insulin per kg of body weight) tolerance tests were performed on 16-h-fasted animals for IPGTT and 5-h-fasted animals for ITT. Glycemia was measured before and at different time after glucose and insulin injections using the Bayer Contour. Circulating insulin levels were measured using the Ultrasensitive Insulin Elisa kit (Mercodia). Oxygen consumption, carbon dioxide production, RER, food intake, and physical activity were measured continuously using Comprehensive Laboratory Animal Monitoring System (CLAMS) consisting of open circuit calorimeter and motion detectors. Body composition was measured by noninvasive quantitative MRI (EchoMRI700). ALT/SGPT Liqui-UV Test (Rate), AST/SGOT Liqui-UV Test (Rate) and Triglycerides LiquiColor™ Test (Mono) Reagents were purchased from Stanbio and used for ALT, AST and triglyceride dosage respectively.

### Histology

Tissues was fixed with paraformaldehyde, paraffin embedded, and sectioned (5 mm) prior to H&E staining. All images were captured with an Axio scan Z1 imager (Zeiss) at 20 × magnification.

### Real-time PCR

Total RNA was extracted from frozen tissues and cells using TRIzol reagent according to the manufacturer’s instructions. RNA concentrations were determined on NanaDrop spectrophotometer. Total RNA (100 ng to 1 mg) was transcribed to cDNA using Maxima cDNA synthesis (Thermo Fisher Scientific). Quantitative real-time PCR was performed on ABI Via detection system, and relative mRNA levels were calculated using comparative threshold cycle (CT) method. SYBR green primers are listed in Table S3.

### Proteomics and phosphoproteomics

eWAT and BAT from HFD and LFD fed mice were lysed in 8 M urea buffer containing protease inhibitors (Sigma) and phosphatase inhibitors (Roche). After brief sonication on ice, the samples were reduced by addition of dithiothreitol (DTT) to a final concentration of 5 mM for 60 min at RT, and alkylated by the addition of iodoacetamide (5 mM) and incubation at RT for 30 min in the dark. Proteins were diluted with 50 mM ammonium bicarbonate to bring urea concentration down to below 1 M and digested with sequence-grade trypsin (1:50 enzyme to protein ratio) at 37 °C overnight followed by the addition of formic acid to 1% in solution. The resulting peptides were desalted using C18 Tips per manufacturer’s instructions (Thermo Fisher Scientific) and subjected to a series of procedures including Peptide Labeling, High pH Reverse Phase Fractionation of Peptides, Titanium Dioxide (TiO2) Enrichment of Fractionated Phosphopeptides and Nanoflow LC–MS as previously outlined^64, 65^.

### Analysis of raw mass spectrometry proteomic data

All acquired MS/MS spectra were searched against the Uniprot mouse complete proteome FASTA database, using the MaxQuant software (Version 1.6.7.0) that integrates the Andromeda search engine. TMT reporter ion quantification was performed using MaxQuant. Enzyme specificity was set to trypsin and up to two missed cleavages were allowed. Cysteine carbamidomethylation was specified as a fixed modification whereas oxidation of methionine and N-terminal protein acetylation were set as variable modifications. For phospho-peptides serine, threonine, and tyrosine phosphorylation were specified as variable modifications. Peptide precursor ions were searched with a maximum mass deviation of 6 ppm and fragment ions with a maximum mass deviation of 20 ppm. Peptide and protein identifications were filtered at 1% FDR using the target-decoy database search strategy. Proteins that could not be differentiated based on MS/MS spectra alone were grouped to protein groups (default MaxQuant settings). A threshold Andromeda score of 40 and a threshold delta score of 8 was applied to phosphopeptides, in accordance with parameters described previously. The MaxQuant output files designated “Phospho(STY)sites” and “ProteinGroups” were used for data normalization and other statistical analysis using in-house generated scripts in the R environment.

### Data analysis and pathway enrichment

Bioinformatic analysis was performed using R: version 4.0. The MaxQuant tables of protein group and phosphosite feature intensities was log transformed and loess normalization was applied. For differential analysis, the LIMMA^66^ R package was used to fit a linear model accounting for the experimental conditions. Moderated t-tests were corrected with the Benjamini–Hochberg method for false discovery rate (FDR). Gene set enrichment analysis was performed using the fgsea R package using gene libraries generated by the Bader lab^67^. Upstream kinase prediction was analyzed using PhosR package^36^ and over-represented phospho-pathways were generated using KEGG database^68^.

### Statistical analysis

Data were analyzed using GraphPad Prism 6.0 software (GraphPad) and are presented as mean ± standard error of mean (SEM). Group comparisons were analyzed using either two-tailed unpaired student t test or a two-way ANOVA followed by multiple comparisons correction method stated in Figure legend. Differences were deemed statistically significant with p < 0.05.

## Supplementary Information

Supplementary Information 1.

Supplementary Information 2.

Supplementary Information 3.
