# Supplementary material for: The cyclin dependent kinase inhibitor Roscovitine prevents diet-induced metabolic disruption in obese mice
Source: Sci Rep. 2021 Oct 13;11:20365. doi: 10.1038/s41598-021-99871-z (PMC8514475; doi:10.1038/s41598-021-99871-z)
Supplement: Supplementary file 1 — Supplementary Information 1. [file 41598_2021_99871_MOESM1_ESM.pdf]

Supplementary Material to:

The cyclin dependent kinase inhibitor Roscovitine prevents diet-induced metabolic disruption in obese mice

Nabil Rabhi[1], Kathleen Desevin[1], Briana Noel Cortez[1], Ryan Hekman[2], Jean Z. Lin[1], Andrew Emili[2] and Stephen R. Farmer[1*]

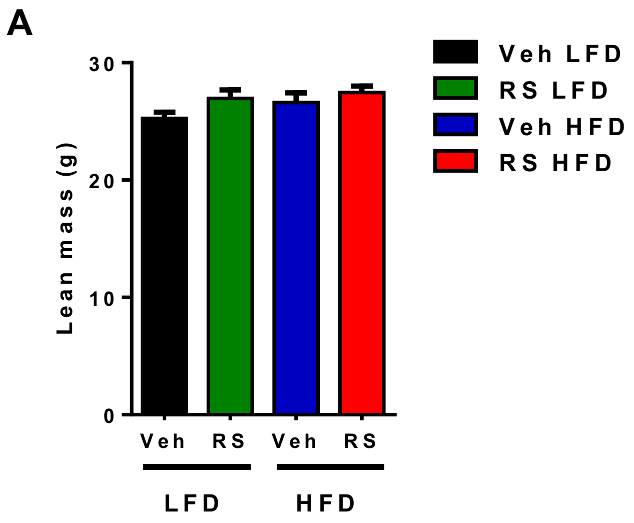

Figure S1: (A) Fat mass determined by nuclear magnetic resonance (NMR) in 19-20 weeks LFD and HFD fed mice treated for 6 weeks with roscovitine or vehicle (n = 5 / group).

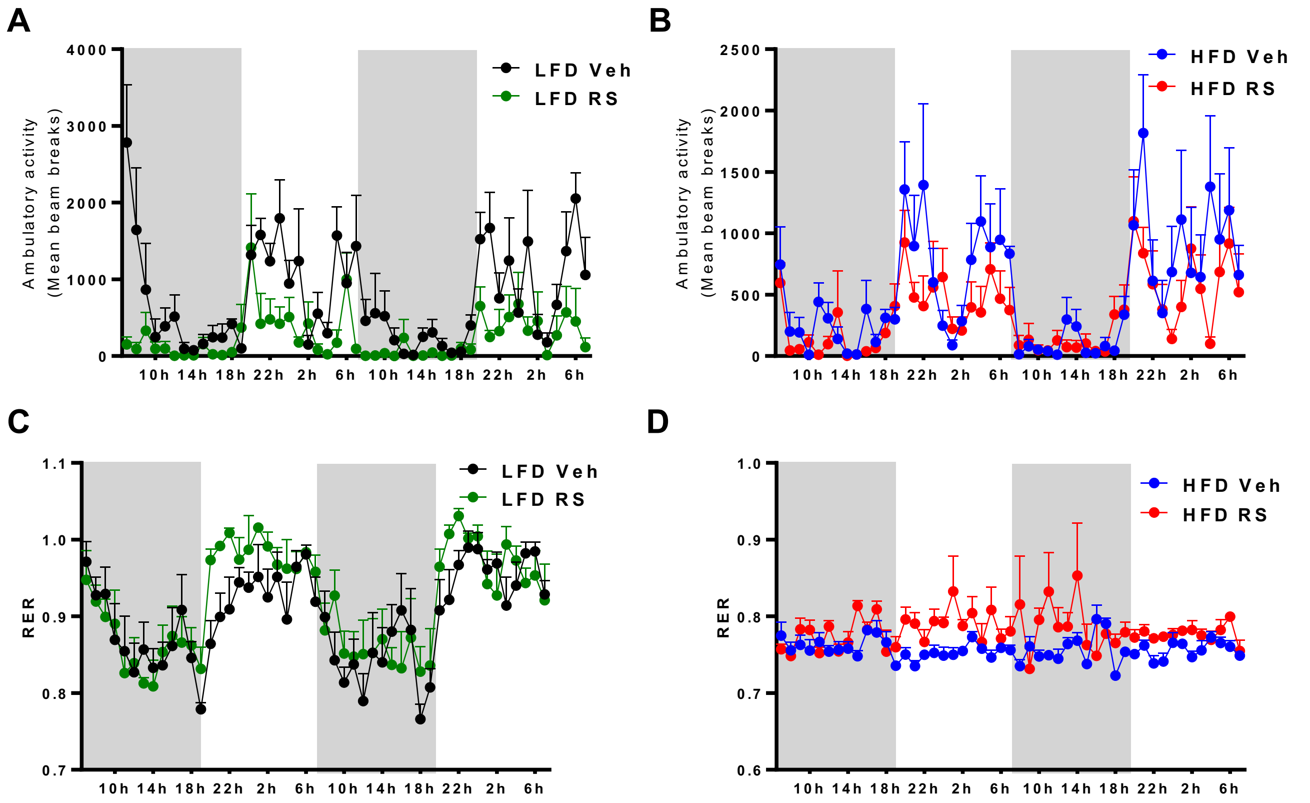

Figure S2: (A, B) Ambulatory activity measured by CLAMS in 19-20 weeks LFD **(A)** and HFD **(B)** fed mice treated for 6 weeks with roscovitine or vehicle (n = 5 / group). **(C, D)** Respiratory exchange ratio (RER) measured by CLAMS in 19-20 weeks LFD **(C)** and HFD **(D)** fed mice treated for 6 weeks with roscovitine or vehicle (n = 5 / group).

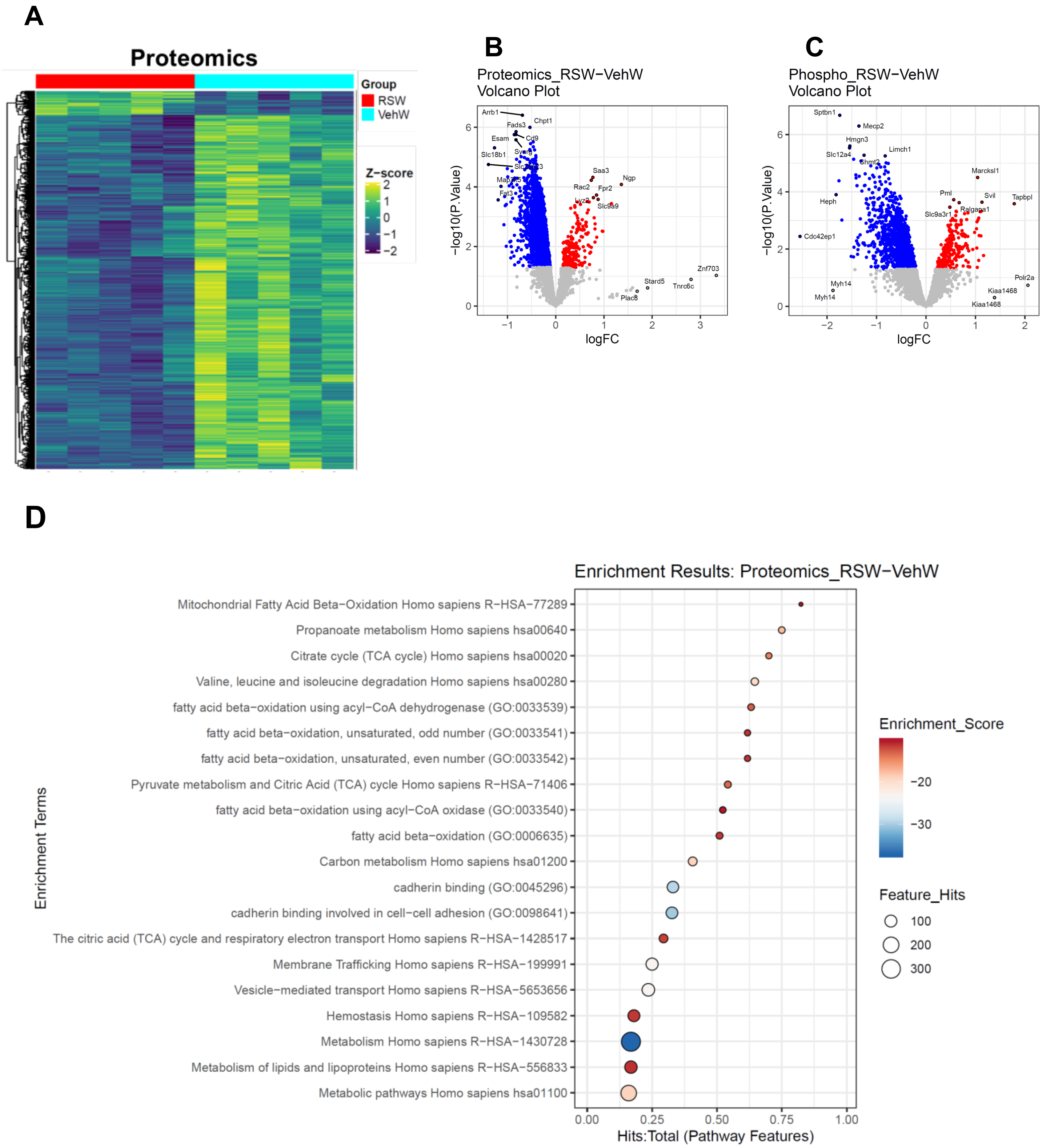
A
Proteomics
Group
RSW
VehW
Z-score
2
1
0
−1
−2
B
Proteomics_RSW−VehW
Volcano Plot
−log10(P.Value)
logFC
Arrb1
Chpt1
Fads3
Cd9
Esam
Slc18b1
Saa3
Ngp
Rac2
Fpr2
Slc9a9
Znf703
Stard5
Tnrc6c
Plac8
C
Phospho_RSW−VehW
Volcano Plot
−log10(P.Value)
logFC
Sptbn1
Mecp2
Hmgn3
Limch1
Slc12a4
Shmt2
Marcksl1
Pml
Svil
Tapbpl
Heph
Slc9a3r1
Ralgapa1
Cdc42ep1
Polr2a
Myh14
Kiaa1468
D
Enrichment Results: Proteomics_RSW−VehW
Mitochondrial Fatty Acid Beta−Oxidation Homo sapiens R−HSA−77289
Propanoate metabolism Homo sapiens hsa00640
Citrate cycle (TCA cycle) Homo sapiens hsa00020
Valine, leucine and isoleucine degradation Homo sapiens hsa00280
fatty acid beta−oxidation using acyl−CoA dehydrogenase (GO:0033539)
fatty acid beta−oxidation, unsaturated, odd number (GO:0033541)
fatty acid beta−oxidation, unsaturated, even number (GO:0033542)
Pyruvate metabolism and Citric Acid (TCA) cycle Homo sapiens R−HSA−71406
fatty acid beta−oxidation using acyl−CoA oxidase (GO:0033540)
fatty acid beta−oxidation (GO:0006635)
Carbon metabolism Homo sapiens hsa01200
cadherin binding (GO:0045296)
cadherin binding involved in cell−cell adhesion (GO:0098641)
The citric acid (TCA) cycle and respiratory electron transport Homo sapiens R−HSA−1428517
Membrane Trafficking Homo sapiens R−HSA−199991
Vesicle−mediated transport Homo sapiens R−HSA−5653656
Hemostasis Homo sapiens R−HSA−109582
Metabolism Homo sapiens R−HSA−1430728
Metabolism of lipids and lipoproteins Homo sapiens R−HSA−556833
Metabolic pathways Homo sapiens hsa01100
Enrichment Terms
0.00
0.25
0.50
0.75
1.00
Hits:Total (Pathway Features)
Enrichment_Score
−20
−30
Feature_Hits
100
200
300

E

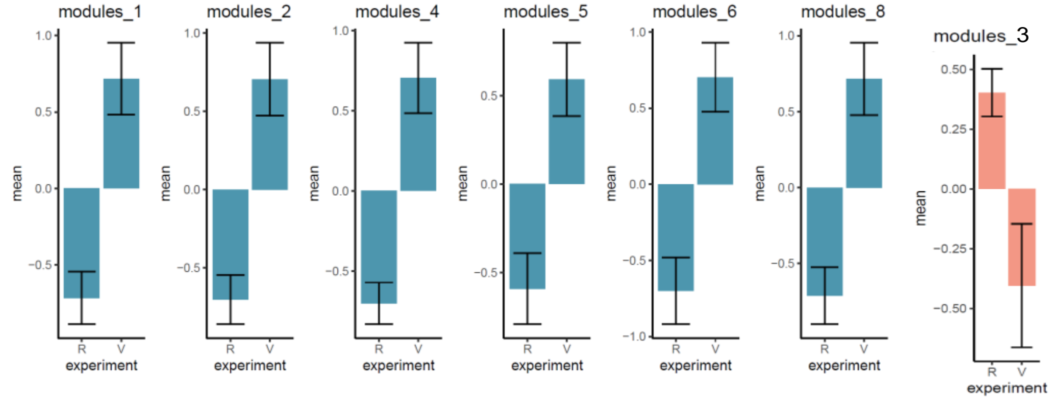

F

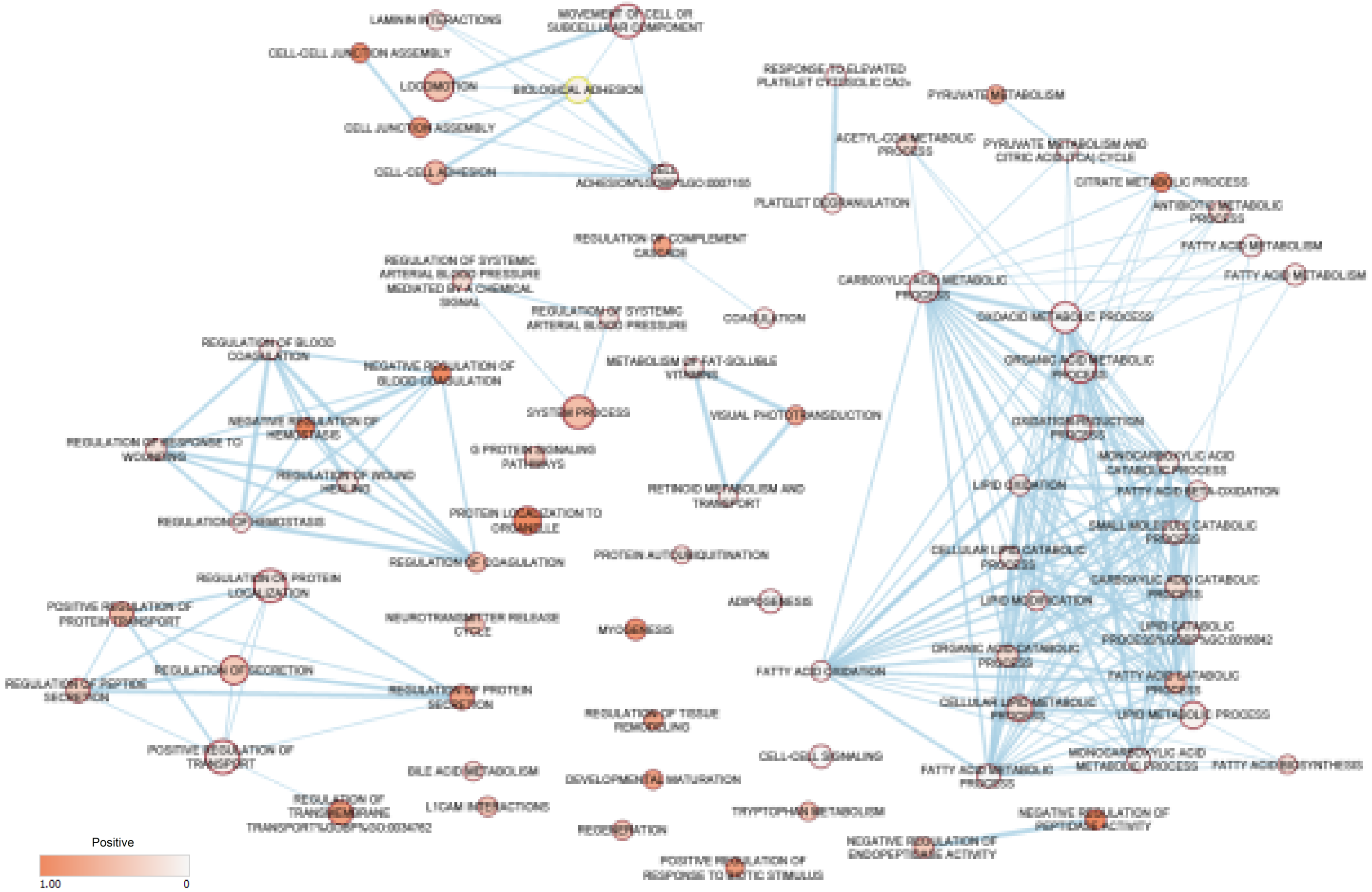

Figure S3: (A) Heatmap of differential proteome in eWAT of 19-20 weeks LFD and HFD fed mice treated for 6 weeks with roscovitine or vehicle (n = 5 / group). **(B)** Volcano plot of up- and down-regulated proteins in eWAT from HFD mice treated with roscovitine compared to vehicle treated. **(C)** Volcano plot of up- and down-regulated phosphoproteins in eWAT from HFD mice treated with roscovitine compared to vehicle treated. **(D)** Dot plot of top enriched Go terms induced in eWAT from HFD mice treated with roscovitine compared to vehicle treated **(E)** Summary of kinase substrates proteins. Bar plots of mean of the phosphosites in each module are summarized for each condition. **(F)** Results of gene set enrichment analysis (GSEA) of up

regulated proteins and phosphoproteins in response to roscovitine treatment in eWAT from HFD mice visualized with Cytoscape Enrichment Map. Each node corresponds to a gene set either up-regulated (red) or down-regulated (blue). Edges (bleu lines) link sets with shared genes, and thickness of lines correlates with the number of genes in common between two sets. Only gene sets with FDR < 0.05 and p < 0.01 were included in visualizations.

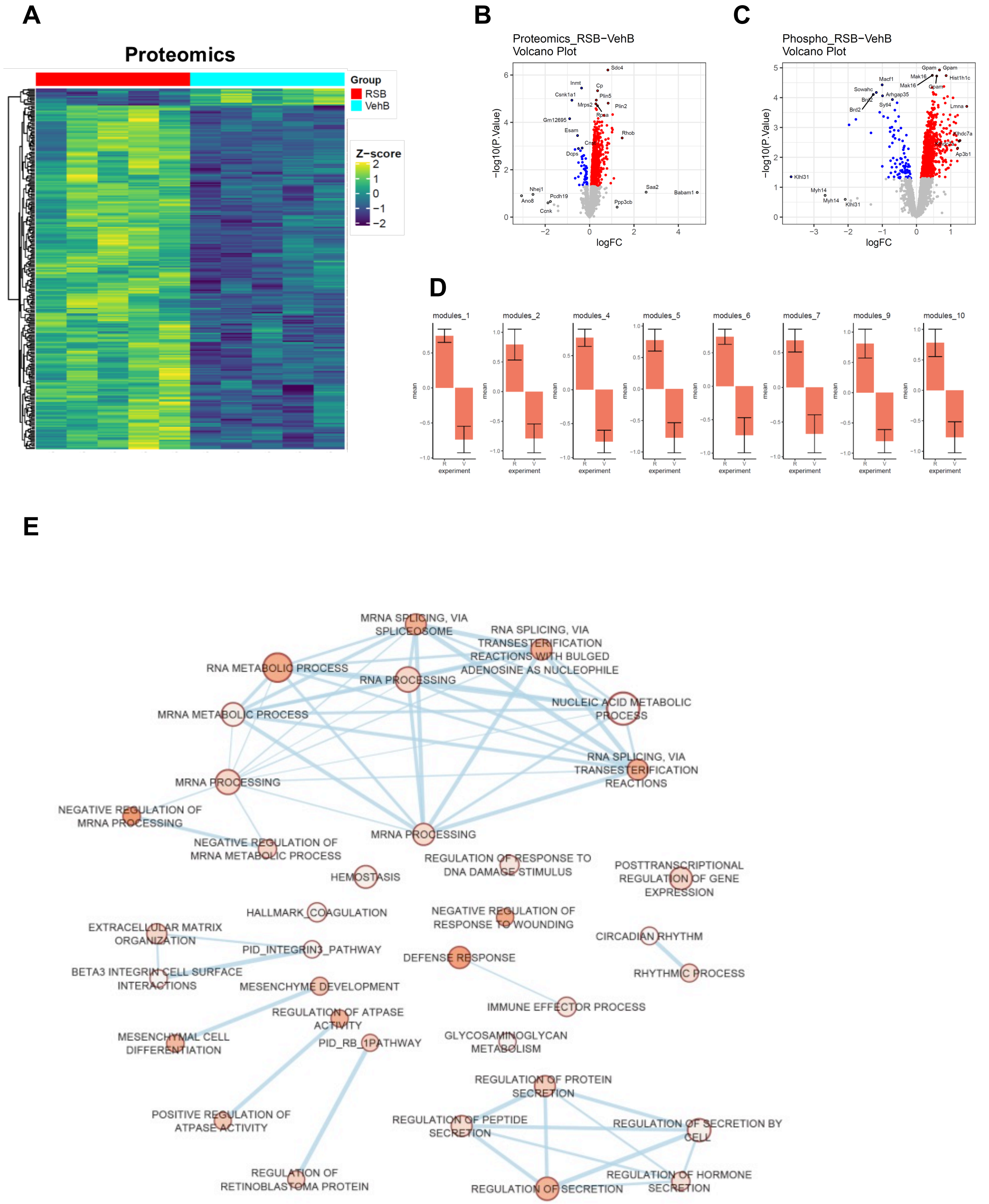
A
Proteomics
Group
RSB
VehB
Z-score
2
1
0
−1
−2
B
Proteomics_RSB−VehB
Volcano Plot
−log10(P.Value)
logFC
Sdc4
Inmt
Cp
Csnk1a1
Plin5
Mrps2
Plin2
Rpsa
Gm12695
Esam
Rhob
Cnp
Dcps
Saa2
Babam1
Nhej1
Ano8
Pcdh19
Ccnk
Ppp3cb
C
Phospho_RSB−VehB
Volcano Plot
−log10(P.Value)
logFC
Gpam
Gpam
Mak16
Hist1h1c
Macf1
Mak16
Sowahc
Gpam
Arhgap35
Brd2
Lmna
Sytl4
Brd2
Klhdc7a
Ap3b1
Klhl31
Myh14
Myh14
Klhl31
D
modules_1
modules_2
modules_4
modules_5
modules_6
modules_7
modules_9
modules_10
mean
experiment
E
MRNA SPLICING, VIA SPLICEOSOME
RNA SPLICING, VIA TRANSESTERIFICATION REACTIONS WITH BULGED ADENOSINE AS NUCLEOPHILE
RNA METABOLIC PROCESS
RNA PROCESSING
NUCLEIC ACID METABOLIC PROCESS
MRNA METABOLIC PROCESS
RNA SPLICING, VIA TRANSESTERIFICATION REACTIONS
MRNA PROCESSING
NEGATIVE REGULATION OF MRNA PROCESSING
NEGATIVE REGULATION OF MRNA METABOLIC PROCESS
MRNA PROCESSING
REGULATION OF RESPONSE TO DNA DAMAGE STIMULUS
POSTTRANSCRIPTIONAL REGULATION OF GENE EXPRESSION
HEMOSTASIS
HALLMARK_COAGULATION
NEGATIVE REGULATION OF RESPONSE TO WOUNDING
EXTRACELLULAR MATRIX ORGANIZATION
CIRCADIAN RHYTHM
PID_INTEGRIN3_PATHWAY
DEFENSE RESPONSE
BETA3 INTEGRIN CELL SURFACE INTERACTIONS
RHYTHMIC PROCESS
MESENCHYME DEVELOPMENT
IMMUNE EFFECTOR PROCESS
REGULATION OF ATPASE ACTIVITY
MESENCHYMAL CELL DIFFERENTIATION
PID_RB_1PATHWAY
GLYCOSAMINOGLYCAN METABOLISM
REGULATION OF PROTEIN SECRETION
POSITIVE REGULATION OF ATPASE ACTIVITY
REGULATION OF PEPTIDE SECRETION
REGULATION OF SECRETION BY CELL
REGULATION OF RETINOBLASTOMA PROTEIN
REGULATION OF HORMONE SECRETION
REGULATION OF SECRETION

Figure S4: (A) Heatmap of differential proteome in BAT of 19-20 weeks LFD and HFD fed mice treated for 6 weeks with roscovitine or vehicle (n = 5 / group). **(B)** Volcano plot of up- and down-regulated proteins in BAT from HFD mice treated with roscovitine compared to vehicle treated. **(C)** Volcano plot of up- and down-regulated phosphoproteins in BAT from HFD mice treated with roscovitine compared to vehicle treated. **(D)** Summary of kinase substrates proteins. Bar plots of mean of the phosphosites in each module are summarized for each condition. **(E)** Results of gene set enrichment analysis (GSEA) of up regulated proteins and phosphoproteins in response to roscovitine treatment in BAT from HFD mice visualized with Cytoscape Enrichment Map. Each node corresponds to a gene set either up-regulated (red) or down-regulated (blue). Edges (bleu lines) link sets with shared genes, and thickness of lines correlates with the number of genes in common between two sets. Only gene sets with FDR < 0.05 and $p < 0.01$ were included in visualizations.

Table S3: SYBR green primers list

Fas F	AGCTGCAACTGTGCAAGGGTCTG
Fas R	TTGCCCAAGCATTGCCGCCTT
Scd 1 F	TTCTTGCGATACACTCTGGTGC
Scd 1 R	CGGGATTGAATGTTCTTGTCGT
Acc 1 F	ATGGGCGGAATGGTCTCTTTC
Acc 1 R	TGGGGACCTTGTCTTCATCAT
Acc2 F	CCTTTGGCAACAAGCAAGGTA
Acc 2	AGTCGTACACATAGGTGGTCC
Col1a1 F	TAAGGGTCCCCAATGGTGAGA
Col1a1 R	GGGTCCCTCGACTCCTACAT
Col3a1 F	CTGTAACATGGAAACTGGGGAAA
Col3a1 R	CCATAGCTGAACTGAAAACCACC
Col6a1 F	GACACCTCTCAGTGTGCTCTGT
Col6a1 R	GCGATAAGCCTTGGCAGGAAATG
Pgc1a F	GAAAACAGGAACAGCAGCAGAG
Pgc1a R	GGGGTCAGAGGAAGAGATAAAG
Dio 2 F	CAGTGTGGTGCACGTCTCCAA TC
Dio2 R	TGAACCAAAGTTGACCACCAG
UCP1 F	TCCTAGGGACCATCACCACCC
UCP1 R	AGCCGGCTGAGATCTTGTTTCC
Cox8b F	GAA CCA TGA AGC CAA CGA CT
Cox8b R	GCG AAG TTC ACA GTG GTT CC
Sma F	GTCCCAGACATCAGGGAGTAA
Sma R	TCGGATACTTCAGCGTCAGGA
Fn F	ATGTGGACCCCTCCTGATAGT
Fn R	GCCCAGTGATTTCAGCAAAGG
Cd 9 F	CTGGCATTGCAGTGCTTGCTA
Cd 9 R	AACCCGAAGAACAATCCCAGC
Mcp1 F	TTAAAAACCTGGATCGGAACCAA
Mcp1 R	GCATTAGCTTCAGATTTACGGGT
Tnfa F	GGTGCCTATGTCTCAGCCTCTT
Tnfa R	GCCATAGAACTGATGAGAGGGAG
F4/80 F	CGTGTTGTTGGTGGCACTGTGA
